# A Narrative Review of Drug Therapy in Adult and Pediatric Cardiac Arrest

**DOI:** 10.31083/j.rcm2406163

**Published:** 2023-06-06

**Authors:** Deborah Jaeger, Alexandra M. Marquez, Marinos Kosmopoulos, Alejandra Gutierrez, Christopher Gaisendrees, Devin Orchard, Tahar Chouihed, Demetri Yannopoulos

**Affiliations:** ^1^Center for Resuscitation Medicine, University of Minnesota Medical School, Minneapolis, MN 55455, USA; ^2^INSERM U 1116, University of Lorraine, 54500 Vandœuvre-lès-Nancy, France; ^3^Division of Cardiology, Department of Medicine, University of Minnesota Medical School, Minneapolis, MN 55455, USA; ^4^Department of Cardiothoracic Surgery, Heart Centre, University of Cologne, 50937 Cologne, Germany; ^5^University of Minnesota Medical School, Minneapolis, MN 55455, USA; ^6^Emergency Department, University Hospital of Nancy, 54000 Nancy, France

**Keywords:** review, cardiac arrest, resuscitation, pharmacology, epinephrine

## Abstract

Drugs are used during cardiopulmonary resuscitation (CPR) in association with chest compressions and ventilation. The main purpose of drugs during resuscitation is either to improve coronary perfusion pressure and myocardial perfusion in order to achieve return of spontaneous circulation (ROSC). The aim of this up-to-date review is to provide an overview of the main drugs used during cardiac arrest (CA), highlighting their historical context, pharmacology, and the data to support them. Epinephrine remains the only recommended vasopressor. Regardless of the controversy about optimal dosage and interval between doses in recent papers, epinephrine should be administered as early as possible to be the most effective in non-shockable rhythms. Despite inconsistent survival outcomes, amiodarone and lidocaine are the only two recommended antiarrhythmics to treat shockable rhythms after defibrillation. Beta-blockers have also been recently evaluated as antiarrhythmic drugs and show promising results but further evaluation is needed. Calcium, sodium bicarbonate, and magnesium are still widely used during resuscitation but have shown no benefit. Available data may even suggest a harmful effect and they are no longer recommended during routine CPR. In experimental studies, sodium nitroprusside showed an increase in survival and favorable neurological outcome when combined with enhanced CPR, but as of today, no clinical data is available. Finally, we review drug administration in pediatric CA. Epinephrine is recommended in pediatric CA and, although they have not shown any improvement in survival or neurological outcome, antiarrhythmic drugs have a 2b recommendation in the current guidelines for shockable rhythms.

## 1. Introduction 

This review aims to give an updated summary of the data available on the main drugs evaluated for cardiopulmonary resuscitation (CPR). Cardiac arrest (CA) is a frequent pathology with an incidence, for out-of-hospital cardiac arrest (OHCA) with attempted resuscitation, of 73 per 100,000 population in the United States and 56 per 100,000 in Europe [1, 2]. Although research on CA has been extensive, the survival rate remains low and stagnant, at approximately 9% [1]. For in-hospital cardiac arrest (IHCA), incidence was 17.16 for 1000 hospital admissions in the United States in 2020, and survival rates were as high as 22.4% [1]. While some interventions like bystander CPR or public defibrillation have had a real impact on outcomes, research on drugs used during CPR has not led to a significant breakthrough in patient care [3, 4, 5].

Over the years, the search for the perfect drug has led to the evaluation of a multitude of different molecules. Despite decades of research and some controversy regarding its benefit, epinephrine remains the only drug strongly recommended for CA since the 1960s. Although some concerns have been raised about possible harmful effects on the microcirculation, epinephrine increases return of spontaneous circulation (ROSC) rates and survival at thirty days when compared to placebo [6]. No other vasopressor drug (e.g., norepinephrine and vasopressin), has shown a significantly improved outcome after CA when compared to epinephrine.

Amiodarone and lidocaine remain the two drugs recommended for shockable rhythms, but in the most recent randomized trial neither of those drugs resulted in a higher survival rate [7]. Neuromodulation using beta-blockers as an adjunctive to antiarrhythmics has shown promising results in small cohorts when antiarrhythmics have failed [8].

Calcium and sodium bicarbonate are widely used in CA patients but are no longer recommended and should be used only in specific circumstances. In fact, there is evidence of harm, with the strongest data in the pediatric population [9, 10].

There is ongoing research exploring a number of investigational drugs for resuscitation. In particular, sodium nitroprusside, a vasodilator, has demonstrated very convincing results with improvement in hemodynamic parameters as well as survival in large animal studies [11].

This state-of-the-art paper on drug therapy in adult and pediatric cardiac arrest provides a comprehensive review of the most up-to-date data regarding vasopressors, antiarrhythmics, frequently used drugs (i.e., steroids, calcium, and bicarbonate), and emerging therapies.

## 2. Vasopressors 

Vasopressors have been used for decades in CA research. The rationale for vasopressor use is to increase coronary perfusion pressure (CPP), which is the main determinant of myocardial perfusion and ROSC. We will review the most commonly used: epinephrine, norepinephrine, and vasopressin.

### 2.1 Epinephrine

#### 2.1.1 Pharmacology and Rationale 

**Fig. 1.**
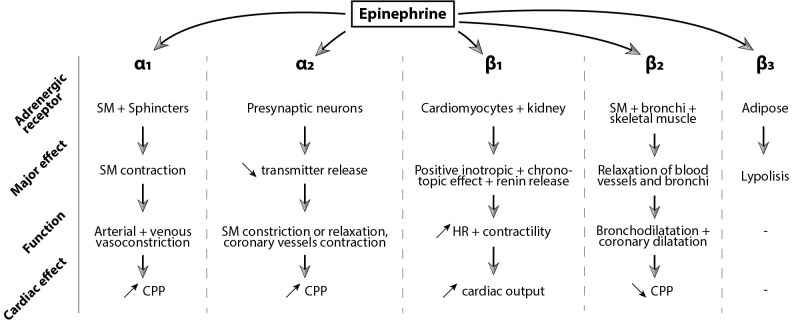
**Epinephrine’s effect mediated by α and ß adrenoreceptors**. SM, smooth muscle; CPP, coronary perfusion pressure; HR, heart rate.

Epinephrine is an adrenergic catecholamine secreted by the adrenal glands in response to a stress stimulus to maintain homeostasis [12]. Its half-life in physiological settings is around 2 to 3 minutes [13]. The key purpose of epinephrine is to achieve ROSC by significantly increasing CPP [14, 15, 16]. Its effect is mediated by α and ß adrenergic receptors. With 1 mg of epinephrine, α and ß adrenoreceptors will be activated, causing vasoconstriction, increasing aortic blood pressure and leading to higher CPP and cerebral perfusion pressure (CePP) [17, 18, 19]. Myocardial perfusion will improve, favoring the onset of ROSC. Epinephrine’s effects are summarized in Fig. 1.

α_1_ receptors are mainly found in the smooth muscle cells of blood vessels and will induce smooth muscle contraction, resulting in peripheral arterial and venous vasoconstriction. α_1_ receptors are also located on cardiomyocytes, mediating contractility and vasoconstriction of the coronary arteries increasing CPP [20]. α_2_ receptors are predominantly present on presynaptic neurons. However, blood flow regulation through α_2_ adrenergic receptors is rather complex, as both pre and post-synaptic receptors play a role and either leads to vasoconstriction or vasodilatation [21]. In the heart, α_2_ receptors are mainly post synaptic on vascular smooth muscle cells and will induce coronary vasoconstriction [20].

ß adrenergic receptors have three subtypes, and their function varies according to the subtype and cell type. ß receptors are more sensitive to epinephrine than α receptors and are activated with lower doses. ${ß}_{1}$ receptors are primarily located in cardiac tissue where they have a positive inotropic and chronotropic effect. ${ß}_{2}$ receptors are predominant in smooth muscle cells of the bronchi and throughout the body. They induce bronchodilatation and smooth muscle relaxation [20, 22, 23]. ${ß}_{1}$ and ${ß}_{2}$ receptors make up 80% and 20% of ß adrenergic receptors expressed in a human heart, respectively. ${ß}_{3}$ receptors have mainly metabolic effects, increasing fat oxidation and improving insulin-mediated glucose uptake but can also have a negative inotrope effect by antagonizing ${ß}_{1}$ and ${ß}_{2}$ effects [24, 25]. Thus, low-dose epinephrine can induce hypotension by activating ${ß}_{2}$ receptors in smooth muscle cells of peripheral arteries. Higher doses of epinephrine will lead to peripheral vasoconstriction and positive inotropic and chronotropic effects.

#### 2.1.2 Epinephrine Dose in Cardiac Arrest 

Since the first CA guidelines in 1974, the American Heart Association (AHA) has recommended a 1 mg dose of epinephrine. This recommendation has persisted for decades, though the grounding evidence is based on a handful of animal studies from the early and mid-1900s. In 1906, Crile and Dolley [26] compared different resuscitation techniques and described the use of 1 to 2 mg of epinephrine in dogs. They found that cardiac compressions associated with ventilation and epinephrine improved survival rates. Later, Pearson and Redding [27, 28] concluded that intracardiac use of 1 mg of epinephrine on an asphyxia dog model of CA improved survival when 9 out of 10 animals survived in the group treated with epinephrine compared to 1 out of 10 in the group treated without epinephrine. Based on these data, 1mg became the recommended dose.

Epinephrine’s α_1_ effects may be dose-dependent, meaning that higher doses of epinephrine may improve CPP and therefore ROSC. Therefore, the idea of higher epinephrine doses during CPR was raised. Soon, the first few animal studies using high-dose epinephrine (HDE) (over 5 mg/bolus) were published and showed some encouraging results with higher myocardial blood flow, CePP, and ROSC [17, 29, 30]. During the 1990s, several double-blinded randomized controlled clinical trials (RCT) compared HDE (5 to 7 mg) to the standard 1 mg dose [31, 32, 33]. They found no significant difference between the HDE and standard-dose groups for survival at one hour (18% *vs.* 23% respectively, *p* = 0.12) and survival to discharge (3% *vs.* 5% respectively, *p* = 0.38) [31]. As summarized in a meta-analysis, HDE was significantly associated with a higher rate of ROSC but not with hospital discharge. Although the physiopathology remains unclear, HDE may worsen the neurological outcome of patients. In 2000, the International Liaison Committee on Resuscitation (ILCOR) formally recommended against HDE.

Clinical studies evaluating doses of epinephrine less than 1 mg are scarce. In 2018, Fisk *et al*. [34] published a before-after study with low (0.5 mg) *vs.* standard epinephrine (1 mg) in OHCA. In the “before” period, paramedics gave 1 mg of epinephrine at 4 min of CPR, followed by an additional 1 mg-dose every eight minutes to patients with a shockable rhythm and 1 mg-dose every two minutes to patients with a non-shockable rhythm. The “after” period used 0.5 mg of epinephrine instead of 1 mg [34]. Survival was not different between groups. This study modified the interval between epinephrine administrations as well as the dose, making it difficult to draw conclusions about the effect of lower doses.

A recent animal study compared low dose epinephrine to the standard 1 mg dose. CPP was significantly lower with a bolus of 0.25 mg compared to 1 mg, whereas with a 0.5 mg bolus CPP was not significantly different [35]. Cerebral near-infrared spectroscopy (NIRS) was used to monitor brain oxygenation and decreased rapidly with higher epinephrine doses. After 32 minutes of CPR, NIRS was 42% (39.5; 59.5) for 0.25 mg; 36% (32; 49) for 0.5 mg, and 32% (29.5; 43.5) for 1 mg (*p* = non-significant). Further studies are needed to assess the effect on cerebral perfusion and neurological outcome comparing low *vs.* standard dose epinephrine.

#### 2.1.3 Time to First Epinephrine Injection 

For OHCA, early administration of epinephrine less than 10 minutes after the 911 call may be associated with improved outcomes, and any delay may be associated with harm [36]. In one study, there was a 10% decrease in the odds of hospital discharge with Cerebral Performance Category (CPC) of 1–2 for every one-minute delay between 911 call and vasopressor administration in OHCA [37]. A meta-analysis showed that early pre-hospital administration might increase the rate of ROSC, survival to discharge and favorable neurologic outcomes [36].

For IHCA, Donnino *et al*. [38] identified a stepwise decrease in survival with increasing time to epinephrine and non-shockable rhythms over 4 minutes. Another study identified that for shockable rhythms, early administration of epinephrine (2 minutes after first defibrillation) was associated with decreased odds of ROSC (*p *< 0.001) and survival with good functional outcome (*p *< 0.001) [39]. It is worth noting that over half of the patients received epinephrine within 2 minutes after the first defibrillation, which goes against current AHA guidelines. In shockable rhythms, use of epinephrine should not delay defibrillation and should be administered after the second electric shock [40]. Non-shockable rhythms have very limited treatment resources outside of epinephrine and benefit more from early administration, especially for OHCA [41, 42, 43].

#### 2.1.4 Intervals for Epinephrine Administration 

Epinephrine administration is recommended every 3 to 5 minutes. However, this recommendation is not based on studies with strong experimental designs according to the AHA [40]. In physiological settings, epinephrine’s half-life is 2–3 minutes with a peak plasma concentration of approximately 90 seconds [44]. Observational studies of OHCA have evaluated intervals between the administration of 1 mg of epinephrine, and the results have been controversial. Grunau *et al*. [45] found improved survival with favorable neurologic outcome when the average epinephrine interval was <3 minutes compared to longer intervals of 5 minutes or more. On the other hand, a recent Japanese observational study enrolling over 10,000 patients with OHCA showed no difference in 1-month neurologically favorable survival when comparing shorter *vs.* longer intervals [46].

#### 2.1.5 Epinephrine’s Effect over Time 

Epinephrine may lose effectiveness with repeated doses over time. During CPR, epinephrine may not have the same metabolism as in a physiological state and could have a longer half-life. A 1 mg dose of epinephrine is high compared to endogenous concentrations of epinephrine, and receptors may be quickly saturated, making subsequent boluses less effective.

In porcine models of CA, CPP increases significantly only after the first few epinephrine injections. In Wagner *et al*.’s study [47], animals were treated with either epinephrine or saline after inducing ventricular fibrillation (VF). Compared to the saline group, animals who received epinephrine had significantly higher CPP for the two first boluses. After the two first injections, CPP still increased but was not significantly different from the saline group [47]. Bar-Joseph *et al*. [48] showed a significant increase in CPP after the first injection of repeated doses of epinephrine (0.1 mg/kg) with an increase of more than 300% and no further increase for the three next boluses. In Jaeger *et al*.’s animal model [35] of VF CA, the CPP absolute increase observed after the first 1mg epinephrine dose was of 93% and decreased with each subsequent dose, down to 46% for the 5th dose.

#### 2.1.6 Epinephrine *vs.* Placebo Studies 

Some researchers have questioned the benefit of epinephrine during CA. In 2011, Jacobs *et al*. [49] published the first RCT comparing epinephrine *vs.* placebo. Although survival in both groups was not significantly different, ROSC rates were significantly higher with epinephrine compared to placebo. A few years later in 2018, Perkins *et al*. [6] published another RCT, the PARAMEDIC-2 study. Over 8000 patients were included to compare the effect of standard-dose epinephrine to placebo [6]. For their primary outcome of survival at 30 days, they found a small but significant improvement with epinephrine (3.2% *vs.* 2.4% respectively; unadjusted odds ratio (OR) = 1.39; 95% CI: 1.06–1.82). Once again, ROSC was higher in the epinephrine group than the placebo group (36.4% *vs.* 11.7%). However, for their secondary outcome of favorable neurologic outcome at 3 months, there was no significant difference (adjusted OR = 1.31; 95% CI: 0.94–1.82). Also, there were more patients in the epinephrine group who survived with severe neurological impairment (mRs score of 4 or 5): 31% (39/126) *vs.* 17.8% (16/90). But, 20 patients were lost to follow-up for neurologic analysis in the epinephrine group and 29 in the placebo group, representing respectively 16% and 32% of the patients surviving until hospital discharge. Moreover, it is important to highlight that the median time from 911 call to epinephrine administration was 21.5 minutes (16–27.3 minutes). Such a duration between collapse and the first drug administration might explain the lack of difference in survival with good neurological outcome. A secondary analysis combining data from those two RCTs showed that the benefit of epinephrine for ROSC was greater for non-shockable rhythms compared to shockable rhythms (OR = 6.52; 95% CI: 5.56–7.63 *vs.* OR = 2.32; 95% CI: 1.86–2.89, *p *< 0.001). Survival with good neurological outcomes was not significantly different between these subgroups [50].

#### 2.1.7 Epinephrine’s Deleterious Effects 

Despite its benefits, epinephrine may also cause harm [50, 51, 52, 53]. Epinephrine improves CPP but also increases vascular resistance by its α_1_ effect, leading to lower coronary flow despite higher CPPs [47]. An identical effect has been identified for cerebral perfusion pressure: epinephrine-induced vasoconstriction may increase perfusion pressure but may not result in higher blood flow or improved oxygenation [14, 54, 55]. The microcirculation may be particularly impaired by epinephrine during CPR. An animal study showed that cerebral cortical microcirculatory blood flow was significantly decreased despite an increase in arterial pressure. Microphotographs in this study demonstrated the disappearance of microvessels of less than 20 μm in the epinephrine-treated group until 5 to 7 minutes after ROSC [54].

Recently, a pediatric CA rat model showed that following CPR with standard epinephrine use, penetrating arterioles showed significant constriction compared to animals resuscitated without epinephrine [56]. The placebo-treated group showed a progressive increase in capillary diameter post-ROSC, whereas capillary diameter remained stable in the epinephrine group [56]. Animal studies demonstrating persisting effects of epinephrine on the cerebral circulation may provide mechanistic evidence for randomized trials that have found decreased favorable neurological survival among patients treated with epinephrine compared to placebo. On the other hand, a pediatric IHCA swine model of asphyxia demonstrated an increase in cerebral blood flow and cerebral tissue oxygenation with the first two doses of epinephrine [57]. Cerebral autoregulation may differ between these pediatric IHCA models and adult OHCA or VF models.

Further epinephrine may increase myocardial oxygen consumption due to ${ß}_{1}$ chronotropy. Animal studies have shown an increased heart rate associated with higher myocardial oxygen consumption, lower oxygen delivery, and impaired myocardial contractility with epinephrine [58, 59, 60]. A regression analysis has also shown that the total epinephrine dose may be associated with post-resuscitation myocardial dysfunction. A total epinephrine dose over 5 mg was significantly associated with a lower left ventricular ejection fraction and higher left ventricular end-systolic diameter [61]. Moreover, epinephrine use may have adverse metabolic effects with increased systemic inflammation and vascular endothelial injury, potentially aggravating reperfusion injury and inducing a systemic inflammatory response [62].

### 2.2 Norepinephrine 

#### 2.2.1 Pharmacology and Rationale 

Norepinephrine (NE) is an endogenous catecholamine that functions as a neurotransmitter in the sympathetic nervous system. NE is mainly an α adrenergic agonist with very little action on ${ß}_{2}$ receptors. However, it is slightly less potent than epinephrine on α receptors in most organs, and its ${ß}_{1}$ agonistic effect is also lower [63]. Therefore, NE has less of a positive chronotropic and inotropic effect, leading to lower myocardial oxygen demand and fewer arrhythmias. Despite these potential advantages, experiments using NE during CPR are scarce.

#### 2.2.2 Norepinephrine in Experimental Studies 

Some experimental studies have shown promising findings when using NE during CPR. NE doses ranged from 0.045 mg/kg to 0.16 mg/kg in the different reported studies [64, 65, 66]. In a VF CA model, NE compared to epinephrine drastically reduced the resuscitation duration from 11.1 ± 3.6 to 1.4 ± 0.3 minutes [64]. Compared to HDE, NE improved myocardial oxygen extraction rate and decreased myocardial lactate [65, 66].

This may be related to the reduced chronotropic effect of NE by lack of ${ß}_{1}$ agonist effect. Another study showed higher myocardial blood flow (118.9 ± 73.1 *vs.* 62.2 ± 45.4 mL/min/100g, *p* = 0.04) with NE compared to HDE but no difference in the oxygen extraction ratio or survival. However, the NE group did demonstrate an increase in oxygen consumption, potentially counterbalancing its positive effects on oxygen delivery [67]. Brown *et al*. [68] evaluated the effect of three different doses of NE (0.08 mg/kg, 0.12 mg/kg, and 0.16 mg/kg) and HDE (0.2 mg/kg) on cerebral blood flow in twenty swine in VF. The two higher doses of NE showed improved cerebral blood flow (CBF) compared to epinephrine (*p *< 0.05). In another swine model of CA in VF, NE significantly improved the cerebral perfusion gradient compared to saline (4.3 ± 1.2 *vs.* 2.5 ± 1.2 kPa respectively, *p *< 0.05) as well as the total brain blood flow (45 ± 21 *vs.* 21 ± 12 mL/min/100 g, *p *< 0.05) but no difference was found between NE and HDE [69]. NE may increase cerebral blood flow dose-dependently. With a stronger α-adrenergic effect compared to epinephrine, it could lead to higher myocardial blood flow and thereby increase CBF.

#### 2.2.3 Norepinephrine in Clinical Trials 

Higher resuscitation rates with NE were found in a small clinical trial of 50 OHCA patients with VF. After the 3rd shock, patients received a blinded treatment of either one dose of 1 mg of NE or 1 mg of epinephrine, followed by epinephrine in both groups if no ROSC was observed. 16/25 patients in the NE group were resuscitated compared to 8/25 in the epinephrine group (*p *< 0.05). Ventricular dysrhythmias were less frequent in the NE group than in the epinephrine group (8/16 *vs.* 6/8, respectively) [70].

Only one pre-hospital RCT has been published comparing high-dose NE to HDE and standard epinephrine [71]. 816 patients were enrolled between 1990 and 1992. Paramedics treated nontraumatic OHCA with up to three doses of either 1 mg or 15 mg of epinephrine or 11 mg of NE. ROSC rates were not significantly different between high-dose NE and standard dose epinephrine (13% *vs.* 8%, *p* = 0.19). Survival rates at hospital discharge were not significantly different between HDE, standard dose epinephrine, and high-dose NE groups (1.7% *vs.* 1.2% *vs.* 2.6%, *p* = 0.83 and *p* = 0.37 respectively). Drawing conclusions about NE from this RCT is challenging and this study should be cautiously interpreted as it is over three decades old, and CA care has changed over time. Moreover, the NE dosage was extremely high (11 mg) compared to HDE.

Taken together, the evidence does not support routine NE use during CPR and requires further investigation. It is not recommended to use NE instead of epinephrine during CPR.

### 2.3 Vasopressin 

#### 2.3.1 Pharmacology and Rationale 

Vasopressin is an antidiuretic hormone secreted by the neurohypophysis. Vasopressin binds two types of receptors, V1 and V2. V1 receptors have a non-adrenergic vasoconstrictor effect in the smooth muscle. This vasoconstrictor effect mainly concerns the renal, musculocutaneous and splanchnic vascular territories. However, its vasoconstrictive effects on coronary and cerebral circulation are theoretically limited and may result in improved myocardial and cerebral perfusion. V2 receptor activation induces an antidiuretic effect by increasing the medullary and cortical permeability of the kidney’s collecting tubule to water. Higher endogenous concentrations of vasopressin have been found in successfully resuscitated patients compared to those who died, arguing that vasopressin may play an important role in achieving ROSC and survival [72]. The effects of vasopressin during CA include increased inotropy and systemic vasoconstriction, and potentiation of catecholaminergic effects [73].

#### 2.3.2 Vasopressin in Experimental Studies 

Vasopressin has been used for decades in animal models of CA, alone or in combination with epinephrine. In both VF and pulseless electrical activity (PEA) models of CA, 0.4 IU/kg of vasopressin significantly improved left ventricular myocardial blood flow (52 ± 8 *vs.* 43 ± 5 mL/min/kg, *p *< 0.01), cerebral blood flow (51 (48–70) *vs.* 18 (10–23) mL/min/100 g, *p *< 0.05) and cerebral oxygenation extraction ratio (0.38 (0.25–0.44) *vs.* 0.47 (0.41–0.57), *p *< 0.05) when compared to HDE [74, 75, 76]. Another VF model also showed improved CPP with a high dose of vasopressin (0.8 U/kg) when compared to standard doses of epinephrine at 2 and 4 minutes after drug administration [77]. Vasopressin’s vasoconstrictor effect lasted longer than epinephrine’s during CPR, up to 30 minutes after drug administration [75, 78]. ROSC rate tended to be more frequent in the vasopressin group compared to placebo, but the difference was non-significant [78].

The effects of vasopressin on survival are more controversial. While some studies showed an increase in survival [74, 79, 80], others found that vasopressin use was not associated with ROSC or survival [77, 81]. As a potent vasopressor, vasopressin may be responsible for lower cardiac index and myocardial contractility during the post resuscitative phase. Indeed, Prengel *et al*. [82] have demonstrated that cardiac index and myocardial contractility were significantly lower during the first 15 minutes after defibrillation in animals treated with vasopressin *vs.* epinephrine.

#### 2.3.3 Vasopressin in Clinical Trials 

None of the major RCTs demonstrated a benefit from the administration of vasopressin alone. A trial including 200 patients with IHCA failed to show any difference between the group treated with an initial first dose of vasopressin (40 IU) and the group treated with epinephrine. In this study, the vasopressin group received epinephrine as a second drug if ROSC did not occur. Rates of ROSC, hospital discharge, and thirty-day survival were similar in both groups [83]. In 2004, an RCT with OHCA found an increased hospital discharge rate among patients with asystole treated with vasopressin instead of epinephrine (4.7% *vs.* 1.5%, *p* = 0.04). Patients were randomized to receive either two doses of 40 IU of vasopressin or two doses of 1 mg of epinephrine, followed by additional epinephrine if no ROSC occurred. Comparing patients who needed additional treatment with epinephrine (after the two first doses of vasopressin or epinephrine), survival to hospital discharge was significantly higher for patients in the vasopressin group regardless of initial rhythm (6.2% *vs.* 1.7%, *p* = 0.002) [84]. This suggested that vasopressin may be more effective if given with epinephrine rather than alone. In 2008, 2894 OHCA patients were randomized in a clinical trial to receive a combination of epinephrine and vasopressin (40 IU) or epinephrine with placebo. There were no significant differences between the combination therapy and the epinephrine-only group in ROSC, survival to hospital admission, or 1-year survival [85]. Finally, a meta-analysis of six RCTs determined that vasopressin did not improve overall rates of ROSC or long-term survival. However, in a subgroup analysis of patients with asystole, vasopressin was associated with higher long-term survival [86].

Unfortunately, the encouraging effects of vasopressin on cardiac and cerebral blood flow shown in experimental studies have not translated to survival or neurologic outcomes in clinical trials. Further research is needed on the mechanisms of vasopressin’s action during CA. The use of vasopressin, whether in place of or in combination with epinephrine, is not recommended during CPR by international resuscitation guidelines.

### 2.4 In Summary

Epinephrine remains the only recommended vasopressor in CA, with a class 1 recommendation. A dose of 1 mg for adults should be administered every 3 to 5 minutes after an intra-venous or intra-osseous line is available in patients with non-shockable rhythms and after defibrillation in those with shockable rhythms [40].

## 3. Antiarrhythmics 

Pulseless ventricular tachycardia (VT) and VF are the most treatable rhythms in OHCA and portend a better prognosis than PEA or asystole [1, 87]. However, a quarter of VT/VF patients have an arrhythmia that is refractory to defibrillation [88]. Antiarrhythmic drugs aim to terminate VT/VF, restore a perfusing rhythm, and achieve sustained ROSC by increasing defibrillation’s rate of success. Although amiodarone and lidocaine have shown an improvement in rate of ROSC and survival to hospital admission, this has not translated to higher neurologically intact survival [7, 89, 90, 91, 92, 93].

### 3.1 Amiodarone and Lidocaine 

#### 3.1.1 Pharmacology and Rationale 

Amiodarone is a class III antiarrhythmic agent. Its main effects are α and ß blocking properties as well as blocking potassium, sodium, and calcium channels [94]. Lidocaine is a class Ib antiarrhythmic agent. It will prolong conduction velocity and shortens the duration of the action potential and the effective refractory period [95].

#### 3.1.2 Amiodarone and Lidocaine in Clinical Trials 

Kudenchuk *et al*. [93] randomized 504 patients with refractory VT/VF arrest to amiodarone *vs.* placebo, finding a higher survival to hospital admission with amiodarone as compared to placebo (44 *vs.* 34%; *p* = 0.03). The Amiodarone *vs.* Lidocaine in Prehospital Ventricular Fibrillation Evaluation (ALIVE) trial compared lidocaine to amiodarone administration in refractory VF arrest in 347 patients [96]. Concordant to the study by Kundenchuk *et al*. [93], amiodarone led to a higher survival to hospital admission (22.8 *vs.* 12.0%; *p* = 0.009) [96]. This study was not powered to detect differences in hospital discharge. Therefore, the Amiodarone Lidocaine or Placebo Study (ALPS) trial was published in 2016. The ALPS trial randomized 3026 patients with OHCA to amiodarone, lidocaine, or placebo and did not find a higher survival to discharge or neurologically intact survival with the antiarrhythmic drugs as compared to placebo [7]. This has been consistent across several systematic reviews and meta-analyses that include the data from the trials just mentioned [89, 90, 91, 92].

Time to treatment is an important determinant of outcomes and may help explain why there has been a lack of association between antiarrhythmic drugs and survival in OHCA [97]. In the ALPS study, there was a significant interaction between witnessed OHCA and survival benefit from antiarrhythmic drugs, with a 5% survival benefit over placebo for both lidocaine and amiodarone in the witnessed OHCA group but no benefit in the unwitnessed arrest group [7]. Time to drug administration is hard to quantify in unwitnessed arrests, and prompt drug administration is difficult in CA trials, with a mean time to medication administration of approximately 19 minutes after emergency medical services activation [7]. A more recent study utilized a Bayesian approach to reanalyze the ALPS data and suggested a 2.9% overall survival benefit for amiodarone (interquartile range (IQR) 1.4–3.8%) and 1.7% for lidocaine (IQR 0.8–3.2%) as compared to placebo [98]. This study also showed that amiodarone offered a strong probability (96%–99%) to improve neurological outcomes in refractory VT/VF patients, higher than for lidocaine or placebo (96%) [98] and as such amiodarone should be strongly considered till further data emerge for clinical use. There is a positive effect with the use of antiarrhythmic drugs as suggested by the consistent trend towards improved survival which may justify its use [92].

Given the potential benefit, current guidelines give amiodarone and lidocaine a 2b indication for refractory VT/VF arrest [43].

### 3.2 Magnesium 

Magnesium does not increase ROSC or survival in VT/VF arrest [90, 99, 100, 101] or undifferentiated rhythms [102, 103]. However, case series have shown its utility in suppressing and preventing Torsades de Pointes [104]. Torsades de Pointes occurs in cases where there is a prolonged QT and often bradycardia leading to, a longer QTc reflective of a prolonged refractory period, in which an early after depolarization occurs, initiating the tachycardia. Magnesium leads to suppression of early afterdepolarizations [105, 106]. Magnesium is not recommended routinely in CA management.

### 3.3 Beta-Blockers 

Patients in CA have high levels of catecholamines, and the activation of ${ß}_{1}$ and ${ß}_{2}$ receptors increases myocardial oxygen demand and potentially worsens ischemic injury. Given the main cause of polymorphic VT is ischemia, ß-blockers are recommended to treat ischemia and have shown that in this setting, they reduce the risk of ventricular arrhythmias [107]. The use of esmolol in combination with epinephrine highlights the role of ${ß}_{1}$ adrenergic effects in myocardial dysfunction. When blocked with esmolol, post-resuscitation contractile function and left ventricular diastolic function improved, and recurrence of VF was reduced compared to epinephrine alone [60, 108, 109]. Esmolol has a protective effect on ischemia and reperfusion injury induced by epinephrine [110]. In patients with CA, the use of esmolol was explored in a small study (N = 25). When compared to placebo, esmolol had a trend towards improved survival though no specific benefit in terms of neurological outcomes [8]. The use of non-selective ß-blockers was found to be superior in terminating electrical storms and recurrence of ventricular arrhythmia, with patients treated with metoprolol and amiodarone being 77% less likely to have a termination of arrhythmic events when compared to those treated with propranolol and amiodarone [111]. More research is needed, and guidelines only make recommendations regarding the use of ß-blockers in the setting of cardiac ischemia [40].

Other antiarrhythmic medications including bretylium tosylate, and procainamide will not be discussed in this review.

## 4. Other Cardiac Arrest Drugs 

We will first present here different drugs evaluated in CA. First, calcium and sodium bicarbonate have been drugs originally recommended in the first edition of the AHA recommendations of 1974. Multiple RCT’s have since shown their lack of benefit. Corticosteroids have been investigated more recently and more specifically in association with vasopressin.

Finally, we will also highlight the promising results of an investigational drug, sodium nitroprusside, over the last few years.

### 4.1 Calcium 

#### 4.1.1 Pharmacology and Rationale 

The use of calcium in CA was recommended by the AHA in their first edition in 1974 [112]. The rationale behind this recommendation was that by increasing the calcium concentration, an ion involved in all muscle cells, contractility of the heart would be increased and defibrillation would be more successful. In healthy hearts, calcium has been shown to increase cardiac index and left ventricular stroke work [113]. During the cardiac cycle, an increase in intracellular calcium concentrations released mainly by the sarcoplasmic reticulum, allow for myocardial contraction.

#### 4.1.2 Calcium in Clinical Trials 

The use of calcium in CA was first described in a case series from 1951 in pediatric cardiac surgery when intracardiac calcium administration resulted in ROSC in four patients [114]. In 1985, two small randomized trials in OHCA showed a higher ROSC rate in the calcium-treated group, although the difference was non-significant [115, 116]. After controversial results from several observational studies [117, 118, 119, 120], a recent multicentric RCT was stopped early due to concern about harm in the calcium group. 383 patients with OHCA were randomized to receive either calcium chloride or placebo after the first dose of epinephrine [121]. 19% had ROSC in the calcium group compared to 27 in the placebo group (*p* = 0.9). At 30 days, 5.2% in the calcium group and 9.1% in the placebo group were alive (*p* = 0.17). Also, among patients with ROSC, 74% in the calcium group had hypercalcemia *vs.* 2% in the saline group. Forty percent of the patients receiving calcium had a calcium lever after ROSC between 1.47 and 2.00 mmol/L vs none in the placebo group. A secondary analysis assessed the long-term outcome of these patients. After one year, 3.6% of patients were alive with a favorable neurological outcome in the calcium group *vs.* 8.6% in the saline group (Relative Risk (RR): 0.42; 95% CI: 0.18–0.97) [122]. A systematic review published in December 2022 including 3 RCTs showed no benefit with calcium administration and possible harm with unfavorable neurologic outcome at 90 days [123]. The possible mechanism behind this finding may be that the anaerobic state of CPR triggers an influx of calcium by the Na/Ca exchanger [124]. Intracellular calcium level rises even more when reperfusion occurs and calcium accumulates in the mitochondria contributing to myocardial cell death [125]. Administering supplemental calcium may induce a calcium overload and hasten cell death.

Calcium is no longer recommended during CPR outside of special circumstances such as hypocalcemia and hyperkalemia, and may be considered in β-blocker or calcium channel blocker overdose [126].

### 4.2 Sodium Bicarbonate 

#### 4.2.1 Pharmacology and Rationale 

Sodium bicarbonate (SB) (${\mathrm{NaHCO}}_{3}$) is a salt that increases pH and corrects metabolic acidosis by utilizing the bicarbonate buffer system [127]. First, it dissociates into sodium and bicarbonate (${\mathrm{HCO}}_{3}$^-^) ions. The bicarbonate ion then binds free hydrogen ions, becoming carbonic acid ($H_{2}$${\mathrm{CO}}_{3}$), which then converts into water and carbon dioxide, which is removed from the body via the respiratory system. The more ${\mathrm{HCO}}_{3}$^-^ ions available, the more H+ ions can be turned into $H_{2}$O, increasing pH [127].

During CA and CPR, no or low systemic blood flow result in a build-up of byproducts of anaerobic metabolism and ineffective removal of carbon dioxide which together lead to systemic acidosis. The harmful effects of severe acidosis include vasodilatation, protein denaturation, impaired ATP production, predisposition to arrhythmias, and a depressed response to vasopressors [128, 129]. The hypothesis of using an alkalizing agent, such as SB, is to buffer hydrogen ions and thus increase the chances of successful resuscitation [130].

The administration of SB during CA was listed as the first-line management in the original 1974 published guideline of Cardiac Care by the AHA [131]. Until the early 1980s, SB was used in about 85% of IHCA [132]. During the following years, evidence arose questioning the beneficial effects of SB.

Adverse effects of SB administration are mainly metabolic alkalosis. There are also concerns for exacerbating hypernatremia and therefore hyperosmolarity and reduced systemic vascular resistance compromising CPP. Also the excessive ${\mathrm{CO}}_{2}$ production could contribute to and increase intracellular acidosis [133, 134].

#### 4.2.2 Sodium Bicarbonate in Clinical Trials 

Several clinical studies have evaluated the use of SB in CA but have reported mixed results [135, 136, 137, 138, 139]. In a prospective, randomized controlled study from 2006, Vukmir *et al*. [140] found a trend toward improved survival rates in patients with prolonged CA (>15 minutes) that did not reach statistical significance and no difference overall in survival rates to hospital admission in patients receiving SB *vs.* placebo. More recently, Kim *et al*. [135] published a retrospective, observational, case-control study and found an association between SB and ROSC within 20 minutes after hospital admission. A smaller study by Ahn *et al*. [136] showed that the administration of SB in prolonged OHCA was not associated with any improvement in survival nor increased neurological outcomes at 1 or 6 months.

Other retrospective studies and meta-analyses have not demonstrated benefit with sodium bicarbonate and have found evidence of possible harm. A study analyzed patients from France and North America. In the North American dataset, 20.6% of patients were treated with SB and the use of SB was associated with a lower likelihood of favorable functional outcomes at hospital discharge. In the French dataset, only 2.2% of patients received SB and SB was not associated with higher survival with favorable neurological outcome [139]. Recently, Alshahrani *et al*. [141] published a meta-analysis investigating the effects of SB in CA, including 14 studies (4 RCT and 10 observational studies) with over 28,000 patients. They showed that SB was associated with poorer rates of ROSC and good neurological outcome at discharge [141].

Since 2010, AHA officially advised against SB use during routine CA except in cases of hyperkalemia or tricyclic antidepressant overdose due to evidence consistently showing outcomes were either the same as or worse than without SB administration [142].

### 4.3 Corticosteroids 

#### 4.3.1 Pharmacology and Rationale 

It is well known that reperfusion after ROSC induces a pro-inflammatory response with a rise in inflammatory biomarkers [143]. A cytokine storm with activation of platelets and leukocytes and vasodilatory shock are common in the post-cardiac arrest syndrome [144, 145]. In other situations, corticosteroids have been shown to reduce the pro-inflammatory response, restore effective blood volume by increasing mineralocorticoid activity, increase systemic vascular resistance, and even improve survival in septic shock [145, 146, 147, 148]. Also, post-resuscitation cortisol production may be compromised because of reperfusion injury to adrenal tissues.

#### 4.3.2 Corticosteroids in Clinical Trials 

For these reasons, it may be logical to consider corticosteroids in the management of CA. However, data from clinical trials have not been convincing. In a recent study, the use of corticosteroids during IHCA CPR was not associated with hemodynamic improvement or lower inflammatory biomarkers as compared to placebo [149]. Very few papers have compared the use of steroids to epinephrine. Apart from an increase in ROSC rate in a nonrandomized, non-blinded study, there was no benefit on outcomes with the use of corticosteroids during CPR [150, 151, 152].

A few RCTs have investigated the use of corticosteroids (40 mg of methylprednisolone) in combination with vasopressin (20 IU) and epinephrine (1 mg) compared to epinephrine alone. Three of them showed an increase in ROSC rate with that combination of drugs [153, 154, 155]. Two also showed an increase in survival to hospital discharge for IHCA, with the most recent study demonstrating a higher survival with favorable neurological outcome [153, 154, 155]. A meta-analysis including those three studies found an OR of 2.09 (95% CI: 1.54–2.84) for ROSC and of 1.64 (95% CI: 0.99–2.72) for favorable neurological outcomes when administering vasopressin with steroids and epinephrine *vs.* epinephrine and placebo [156]. A more recent study published in 2022 confirmed these findings that the addition of corticosteroid and vasopressin after the first dose of epinephrine improved rate of ROSC but had no effect on long-term survival when compared to epinephrine alone [157].

In summary, the use of corticosteroids during CPR may be considered (recommendation 2b) but has unsure benefits.

### 4.4 Sodium Nitroprusside 

#### 4.4.1 Pharmacology and Rationale 

Sodium nitroprusside (SNP) has emerged as a potential paradigm shift in the medical management of CA. While traditional pharmacologic approaches with vasopressors rely on improving systemic pressures in order to improve the CPP, SNP is a potent vasodilator that results in both large and small vessel relaxation through the release of nitric oxide [158]. Indeed, the ischemia-reperfusion injury resulting from the low-flow state of CA and CPR is linked to marked endothelial dysfunction. Troelsen *et al*. [159] have demonstrated that coronary arteries in rats suffering from CA have reduced vasodilatory capacity after being challenged with acetylcholine when compared to controls. Moreover, the concentration of vascular adhesion molecules like P-selectin and von-Willebrand factor is increased after CA [159]. Animals suffering from CA experience marked decreases in the neuronal nitric oxide synthase levels [160, 161] and thus a limited capacity for nitric oxide-mediated vasodilation and blood flow regulation. Knock-out of the nitric oxide synthase gene before the induction of CA is also associated with worse outcomes, including a decreased ROSC rate and worse left-ventricular function [162]. These findings suggest a potential benefit in CA from medications targeting nitric oxide like SNP.

#### 4.4.2 Sodium Nitroprusside in Experimental Studies 

This hypothesis has been successfully validated and replicated in multiple animal studies using the porcine model of CA. SNP-enhanced CPR has been shown to markedly improve carotid blood flow in a porcine model of CA when compared to epinephrine. In addition, SNP treatment improved the metabolic derangement observed in CA, as these animals had improved arterial pH [163, 164]. Left ventricular diastolic function after ROSC appears to also be improved as animals treated with SNP was shown to have decreased left ventricular septal wall thickness and increased cavity diameter [165]. Cerebral histology from SNP-treated CA animals demonstrated decreased ischemic brain injury [166]. These findings were translated to increased 24-hour survival and improved neurologic status and cerebral performance capacity [11, 163, 164, 165, 167].

While SNP is generally considered to be anti-hypertensive, systemic blood pressures were maintained in these experiments. SNP exerts its effects on both the systemic and pulmonary vasculature as it has been demonstrated to reduce hypoxic pulmonary vasoconstriction and thus augment the left ventricular preload and CPR cardiac output. Hypoxic vasoconstriction reversal leads to intrapulmonary shunts and concomitant increases in the alveolar-arterial oxygen gradient. The resulting hypoxia was shown to respond to supplemental oxygen [168]. Vasodilation is also observed in the cerebral microvasculature, as SNP-treated animals had an increased number of perfused microvessels, greater arteriolar diameter, and a higher microvascular flow index [169]. The hemodynamic effects of SNP are observed in animals receiving either conventional [11, 166, 167] or extracorporeal CPR [168]. These findings lay the framework for a clinical trial design testing SNP in human CA patients.

Recommendations of drugs for adult CA are summarized in Table 1 with their respective class of strength and level of evidence (Table 2).

**Table 1.** **Drug administration for adults during CPR**.

Drug		COR	LOE	Recommendation
Epinephrine		1	B-R	Use is recommended in CA
	- 1 mg every 3–5 min	2a	B-R	Reasonable to administer 1 mg every 3–5 minutes
Norepinephrine			No recommendations	
Vasopressin		2b	C-LD	May be considered alone or with epinephrine but offers no advantage as substitute for epinephrine
Amiodarone		2b	B-R	May be considered for VF or pVT if unresponsive to 3 defibrillations
Lidocaine		2b	B-R	
Magnesium		3	B-R	Routine use not recommended-no benefit
Beta-blockers			No recommendations	
Calcium		3	B-NR	Routine use not recommended-no benefit
Sodium Bicarbonate		3	B-R	Routine use not recommended-no benefit
Sodium Nitroprusside			No recommendations	
Corticosteroids		2b	C-LD	Use during CPR is of uncertain benefit

COR, Class of recommendation and LOE, level of evidence (adapted from 2020 AHA guidelines for cardiopulmonary resuscitation and emergency cardiovascular care, Circulation, 2020). The colors of the drug correspond to the class of recommendation (Please see Table 2). CA, cardiac arrest; VF, ventricular fibrillation; pVT, pulseless ventricular tachycardia; CPR, cardiopulmonary resuscitation; AHA, the American Heart Association.

**Table 2.** **Class of recommendation and level of evidence**.

Class of recommendation:	Level of evidence:
**1**: Strong recommendation, benefit >>> risk	**B-R**: randomized, moderate quality evidence from 1 or more randomized controlled trial
**2a**: Moderate recommendation, benefit >> risk	
**2b**: Weak recommendation, benefit ≥ risk	**B-NR: nonrandomized, moderate quality evidence from 1 or more well-designed, well executed nonrandomized, observational or registry studies**
**3**: No benefit, benefit = risk or harm	**C-LD: limited data, randomized or nonrandomized observational or registry studies with limitations of design or execution**

## 5. Pediatric Cardiac Arrest 

Drugs are used during pediatric CA to improve CPR hemodynamics, restore a perfusing rhythm, and address reversible causes. Because children have distinct differences in physiology and reasons for arrest compared to adults, it is worthwhile to review pediatric-specific literature. It is important to note that the evidence in pediatrics is primarily derived from IHCA. Guidelines are summarized in Table 3.

**Table 3.** **Drug administration during pediatric cardiac arrest during CPR**.

Drug	COR	LOE	Recommendation
Epinephrine	2a	C-LD	It is reasonable to administer epinephrine. IV/IO route preferred to ETT
			It is reasonable to administer initial dose within 5 minutes
			It is reasonable to administer epinephrine every 3–5 minutes
Amiodarone	2b	C-LD	Amiodarone or lidocaine may be used for shock resistant VF or pulseless VT
Lidocaine			
Sodium Bicarbonate	3: HARM	B-NR	Routine administration is not recommended (in the absence of hyperkalemia or sodium channel blocker toxicity)
Calcium	3: HARM	B-NR	Routine administration is not recommended (in the absence of hypocalcemia, hyperkalemia, hypermagnesemia, or calcium channel blocker overdose)

COR, Class of recommendation and LOE, level of evidence (adapted from 2020 AHA guidelines for cardiopulmonary resuscitation and emergency Cardiovascular care; Circulation; 2020). The colors of the drug correspond to the class of recommendation (Please see Table 2). IV, intra-venous; IO, intraosseous; ETT, endo-tracheal tube; VF, ventricular fibrillation; VT ventricular tachycardia; AHA, the American Heart Association.

### 5.1 Epinephrine 

Epinephrine is the pharmacologic cornerstone of pediatric CPR and has been implemented in resuscitation for over 100 years [26]. However, there are controversies and knowledge gaps regarding timing, dose and dosing intervals, and long-term outcomes [170].

The strongest evidence for timing of the initial dose comes from a series of “Time to Epinephrine” studies using the AHA Get With the Guidelines registry. For pediatric patients IHCA with initially non-shockable rhythms, Andersen *et al*. [171] showed that earlier epinephrine (within the first 2 minutes) was associated with ROSC, higher survival to discharge, and neurologically favorable survival. Delays to first epinephrine linearly decreased the chance of a good outcome.

What dose should be given for pediatric CA? Perondi and colleagues [172] conducted a randomized, double-blinded trial of standard (0.01 mg/kg) *vs.* high dose (0.1 mg/kg) epinephrine, administered as a rescue after a single failed dose of standard epinephrine. The trial found no benefit and possible harm with high doses [172, 173]. Lower doses of epinephrine are often used and may be beneficial during the peri-arrest period, but there is no data to support lowdose epinephrine during CPR [174, 175].

How often should epinephrine be given? Current guidelines are grounded by a practical approach that allows providers to sync epinephrine doses with pulse checks, compressor changes, and defibrillation. There are no randomized trials, and observational data are conflicting regarding epinephrine dosing intervals [176, 177]. Kienzle *et al*. [176] used documented epinephrine times taken directly from code sheets. In her study, a quarter of patients received “frequent” epinephrine (given every ≤2 minutes), and these patients had higher ROSC and favorable neurologic survival [176]. This was a time-mediated phenomenon whereby the beneficial effect of frequent epinephrine was largely mediated by shortening the duration of CPR. Further supporting this finding, the ICU-RESUS trial, a hybrid cluster-randomized interventional trial across 18 US pediatric intensive care units (ICUs) and cardiovascular intensive care units (CVICUs) comprising a novel patient-centric CPR improvement bundle, also found that frequent epinephrine use was associated with improved outcomes in children with IHCA [178].

For now, guidelines for epinephrine use in pediatric patients with CA are weak to moderate recommendations based on limited data [170, 179, 180] (Table 3). To summarize, “it is reasonable” to administer 0.01 mg/kg of epinephrine during CA, with the first dose given within 5 minutes or as early as possible and subsequent doses in 3–5 minutes intervals. Further study is needed to understand the impact of epinephrine on long-term outcomes.

### 5.2 Anti-Arhythmics 

The use of amiodarone and lidocaine for shock-resistant VF and pVT in children with CA should not be extrapolated from adult data, because the etiologies of pediatric dysrhythmias (i.e., congenital heart disease, inherited conduction abnormalities, myocarditis, and cardiomyopathies) differ from those in adults (primarily coronary artery disease) [181]. The evidence in pediatrics is limited to two observational cohorts of IHCA from the Get With the Guidelines registry [182, 183]. In brief, there were no differences in survival to hospital discharge or favorable neurologic outcomes among children receiving amiodarone or lidocaine for a shockable rhythm.

### 5.3 Calcium and Sodium Bicarbonate 

Calcium and sodium bicarbonate are commonly used during pediatric CA but are not recommended outside of specific scenarios due to their association with harm [170, 179]. Secondary analyses of the recent multicenter ICU-RESUS trial found that sodium bicarbonate and calcium were used in 40–50% of all CA and were each independently associated with mortality and poor neurologic outcome [184, 9]. Registry data similarly found that calcium and bicarbonate were each associated with worse outcomes, longer duration of CPR, greater illness severity, and use of other advanced life support interventions [10, 185]. Although there is biological plausibility that calcium and bicarbonate could cause harm, it remains unclear whether these drugs have a true treatment effect or are simply associated with “last ditch” efforts in challenging resuscitations.

## 6. Conclusions 

Despite many remaining gaps, epinephrine, and to a lesser extent amiodarone and lidocaine, stand as the only drugs recommended during resuscitation by international guidelines. Epinephrine administration remains recommended every 3 to 5 minutes at a standard dose of 1mg for adults and 0.01 mg/kg for pediatric CA. Although routinely used, calcium and sodium bicarbonate are not recommended except for specific circumstances and may cause harm. Further investigations are needed regarding the use of other vasopressors, beta-blockers, or sodium nitroprusside during CPR in clinical settings.
